# Seasonal estimation of groundwater vulnerability

**DOI:** 10.1038/s41598-023-36194-1

**Published:** 2023-06-15

**Authors:** Adrian I. Cervantes-Servin, Meenakshi Arora, Tim J. Peterson, Vincent Pettigrove

**Affiliations:** 1 Department of Infrastructure Engineering, The University of Melbourne, Parkville, Victoria 3010 Australia; 2 Department of Civil Engineering, Monash University, Clayton, Victoria 3800 Australia; 3 Aquatic Pollution Prevention Partnership, Royal Melbourne Institute of Technology, Melbourne, Victoria Australia

**Keywords:** Environmental chemistry, Hydrology, Civil engineering

## Abstract

Index-based methods estimate a fixed value of groundwater vulnerability (GWV); however, the effects of time variations on this estimation have not been comprehensively studied. It is imperative to estimate a time-variant vulnerability that accounts for climatic changes. In this study, we used a Pesticide DRASTICL method separating hydrogeological factors into dynamic and static groups followed by correspondence analysis. The dynamic group is composed of depth and recharge, and the static group is composed of aquifer media, soil media, topography slope, impact of vadose zone, aquifer conductivity and land use. The model results were 42.25–179.89, 33.93–159.81, 34.08–168.74, and 45.56–205.20 for spring, summer, autumn, and winter, respectively. The results showed a moderate correlation between the model predictions and observed nitrogen concentrations with R^2^ = 0.568 and a high correlation for phosphorus concentrations with R^2^ = 0.706. Our results suggest that the time-variant GWV model provides a robust yet flexible method for investigating seasonal changes in GWV. This model is an improvement to the standard index-based methods, making them sensitive to climatic changes and portraying a true vulnerability estimation. Finally, the correction of the rating scale value fixes the problem of overestimation in standard models.

## Introduction

Groundwater is a vital resource in many countries around the world as an important source of freshwater supply to meet growing agricultural and urban demands; in some cases, groundwater can be the limiting factor for agricultural and natural production. In recent decades, groundwater has become polluted due to the excessive use of agrochemicals and other sources of contamination. In Victoria, Australia, some studies have shown the occurrence of the pesticides atrazine, simazine chlorpyriphos, and DDT in groundwater^1–15^. Groundwater vulnerability (GWV) assessment can be defined as the assessment of the contamination risk of aquifers from anthropogenic pollution, where the results are represented as vulnerability maps. However, standard index-based methods for estimating GWV have not evolved since 1985^16^. A major limitation of currently used approaches is their static nature, as the estimations do not account for seasonal variations. In this study, we concentrate on improving such methods by introducing a time-variant estimation of GWV that is able to reflect varying hydrogeologic and hydroclimatic conditions.

Existing index-based methods for estimating GWV produce spatially fixed values, as they consider vulnerability to be static in time and space^17,18^. Such methods fail to depict changes caused by climatic variations, land use and impacts to aquifers, such as water pumping during the year^19–22^. Hence, pollutant concentrations could vary temporally within the same aquifer. Groundwater net recharge is a spatially and temporally dynamic factor that varies throughout the year. Another dynamic factor is the depth to the water table; as a result of changes in climatic conditions throughout the year, groundwater depth can change significantly.

Index-based methods are most commonly utilized to estimate GWV due to their ease of use and lower data requirements^23–29^. Pesticide DRASTIC is a variant of the standard index-based method that accounts for specific processes that affect the fate and transport of pesticides to the water table^30^. Among more than thirty index-based methods^29,31–34^ the most commonly used are AVI, DRASTIC, GOD, EPIK, SINTACS, SEEPAGE, and ISIS^28,31^. Index-based methods are grounded on two different scale components, weights and ratings, that produce a vulnerability score. The first component comprises seven hydrogeological factor weights (w) with values ranging from 1 to 5. These values represent the importance of each factor compared to the others. The second component is the factor rating (r), with values ranging from 1 to 10 for each factor. The rates represent the importance of the range of values or types of media for each factor^30^.

Groundwater vulnerability is classified as intrinsic and specific^31,32,35^. Intrinsic GWV is based solely on the hydrogeological and geomorphological features of the landscape^32,36^, and specific GWV is referred to as the vulnerability that a pollutant or group of pollutants pose to a specific area^31,36^. Water contamination correlation models are complex^37,38^, and this complexity is further magnified in groundwater systems. Standard GWV methods produce a fixed estimation of vulnerability and fail to incorporate temporal and spatial changes due to climate variations. Index-based methods have been criticized because factor weights may not represent the actual hydrogeological characteristics of the study area, and the models produce a result that may underestimate or overestimate the GWV. Additionally, Pacheco et al.^37^ pointed out the need to overcome weighting value subjectivity and identified different approaches to address factor weighting: (1) single factor sensitivity analysis, (2) nonlinear Spearman correlation analysis, (3) logistic regression, (4) minimization of factor redundancy by correspondence analysis and (5) the standard DRASTIC-Delphi (consensus) approach. Additionally, although the analytical hierarchy process (AHP) is a widespread tool^39–41^, AHP weighting techniques are not explicitly designed for the adjustment of factor weights. The correspondence analysis (CA) tool identifies the real weight in correlated variables and has been shown to be the best method to address subjectivity when assigning weight values^25,37^.

This study presents a novel approach that accounts for both temporal and spatial changes, making the estimation of GWV more accurate. We used a modified Pesticide DRASTIC^42^ model as groundwork and added the factor land use (L) as per Alam et al.^43,44^. This model was applied to the Glenelg Hopkins Region in Victoria, Australia. The ranges used in this new model are different from the original DRASTIC model, because Australia’s hydrogeological conformation is different from that of America and Europe. First, we separated static factors (A, S, T, I, C and L) from dynamic factors (D and R). Second, we introduced a double correspondence analysis that allows the model to derive weights that account for temporal variability. Groundwater recharge (R) and depth (D) are the factors responsible for the temporal and climatic changes in the time-variant Pesticide DRASTICL GWV index. As a result, the model produces an independent estimation of GWV for each season: winter, spring, summer and autumn.

## Materials and methods

### Study area

**Figure 1:**
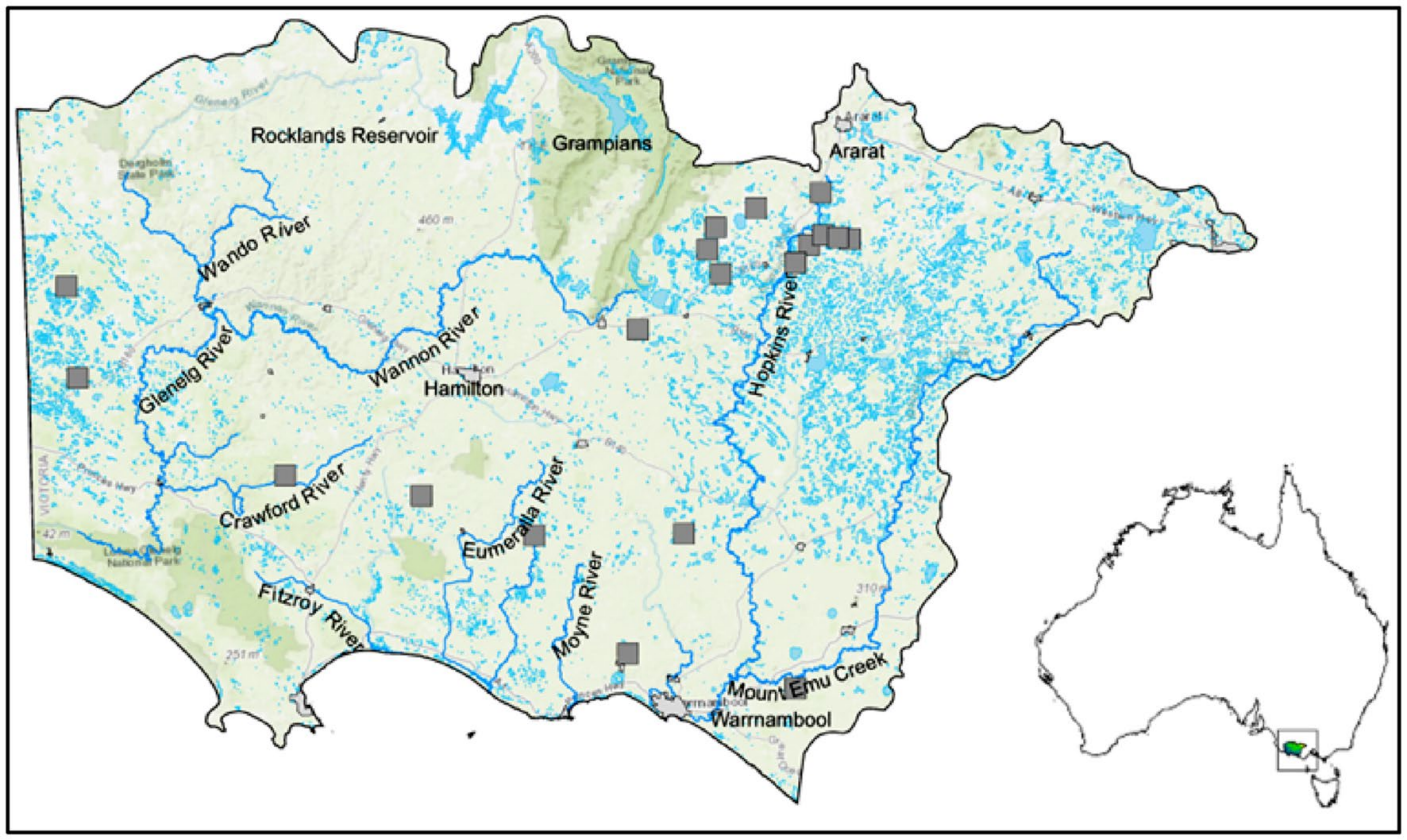
Glenelg Hopkins Catchment Management Authority in Victoria, Australia. Grey squares are monitoring bores for groundwater pollution.

The study area covers the Glenelg Hopkins (GH) region in the western part of Victoria State in Australia (Fig. 1). The region encompasses 2.67 million hectares that extend west from Ballarat to the South Australian border and south to the coast. It comprises 12% of the total area of Victoria^45^. The north is dominated by the Grampians, the Dundas and Merino Table Lands, and the West Victorian Uplands, with the flatter Volcanic Plains characterizing the south. The slope is prominent in the Grampian region, ranging from 100 down to 0% in the Volcanic Plains, with an average slope of 3.48% in the rest of the territory. Maps were produced using ArcMap 10.4.1 https://desktop.arcgis.com/en/arcmap/10.4/get-started/setup/arcgis-desktop-quick-start-guide.htm

Warrnambool and Casterton are the two major towns in the GH region. The mean annual maximum temperature in Warrnambool and Casterton is 19.2 °C and 20.1 °C, with a high of 38.3 °C and 38.5 °C in 2017, respectively. The mean annual minimum temperature in Warrnambool and Casterton is 8.9 °C and 8.4 °C, and the lowest in 2017 was −1.0 °C and −3.0 °C, respectively (BoM http://www.bom.gov.au/climate/averages/maps.shtml, accessed March 2018). There are two distinct climates: Csb is temperate with dry and warm summers, and Cfb is temperate with dry winters and warm summers^46^.

The hydrological subregions are the Hopkins River Basin (East), which includes the Hopkins and the Merry rivers; the Portland Coastal (Southcentral), which includes the Moyne, Eumeralla, Fitzroy and Surry rivers; and the Glenelg River Basin (Northcentral and West), which includes the tributaries of the Glenelg River. The largest water body in the basin is the Rocklands Reservoir^45^.

**Figure 2:**
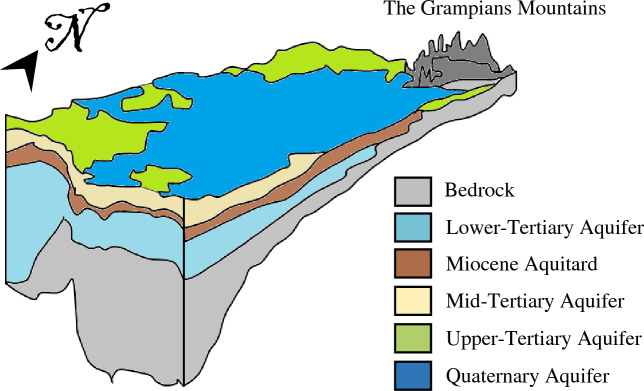
Cross-section of the hydrogeological units in the Glenelg Hopkins region.

Different soil lithologies are present in the region, with the most abundant lithologies including basalts, sedimentary, duricrust, and aeolian, followed by alluvium, limestone, alluvial, granite, colluvial, lacustrine/aeolian, and volcanic. The least abundant soils are fluvial, aeolian and lagoonal^45^. The hydrogeological features of the study area can be seen in Fig. 2.

### Methods

We developed a novel method comprising five steps: (1) Data preparation. (2) Calculation of the Pesticide DRASTICL vulnerability index (IDL) using dynamic factors (D and R) and static hydrogeological factors (A, S, T, I, C, and L). (3) Independent computation of the correspondence analysis (CA) for both dynamic and static factor groups. (4) Upscaling the resulting eigenvalues to the Pesticide DRASTICL weight scale (1 to 5). (5) Recalculation of the seasonal IDL using the calculated weights from step 4.

The sources of data used for Pesticide DRASTICL factors are summarized in Table 1. Both dynamic factors were estimated with models: for D, we used HydroSight^39^, and for R, we used data resulting from the groundwater transient model for the Glenelg Hopkins Catchment Management Authority (CMA). The transient model included a three-stage approach: pre-development—1985, calibration—1985 to 1994, verification—1995 to 1999 and post-development—1995.

**Table 1:** Sources for data used for Pesticide DRASTICL modelling.

Factor		Description
Seasonal depth	(D_s_)	This factor was computed from the average monthly values in each season from 1980–2014 as per Peterson and Wester^47^
Seasonal net recharge	(R_s_)	This factor was calculated as the average of monthly values within each season from 1985–1995. Data were acquired by request to DELWP https://www2.delwp.vic.gov.au/. R_s_ values are resulting values from the “Glenelg Hopkins CMA Groundwater Model”^45, 48^
Aquifer media	(A)	This factor was derived from the GWFS map coupled with the type of consolidated material of the top layer aquifer^49^
Soil media	(S)	Data were accessible from the Victorian Soil Atlas. Soil media does not reflect depths of soil and soil mixtures in the horizontal horizon. It is the uppermost layer of the soil profile
Topography slope	(T)	This factor was calculated in ArcMap10.4.1 from the Victoria Digital Elevation Model^50^
Impact of vadose zone	(I)	This factor was assembled from the GWFS and aquifer material were taken from aquifer descriptions in the Groundwater Framework Report^48^
Hydraulic conductivity	(C)	The conductivity of the aquifer data was obtained from the 2002 Groundwater Flow Systems – GH CMA report^48^ and corrected with the GH groundwater transient model^45^
Land use	(L)	Data are available from the (VLUIS)^51^

#### Computation of the Pesticide DRASTICL vulnerability index (I_DL_)

The Pesticide DRASTICL index is calculated summing the weight of all factor weight values times their corresponding rating value as presented below:

1 $$ I_{PDL} = \mathop \sum \limits_{1}^{i} r_{i} *w_{i} $$

where I_PDL_ = Pesticide DRASTICL index (dimensionless).w = factor weight (dimensionless)r = rating for the corresponding range (dimensionless)

The modified weighting factor values are shown in Table 2. Weights represent how important each factor is when compared to others and range from 1 to 5, with 5 being the most significant factor and 1 the least significant factor^30^. Each factor was reclassified by assigning the corresponding rating value and converted to raster format using ArcMap10.4.1. Each factor layer was portrayed in a grid of 50 m × 50 m using a georeferenced GDA 1994 Lambert Conformal Conic projection. Ranges represent the significant media type that contributes to the pollution potential, and such values change spatially and within their own scale^30^. Every range is evaluated against each other to calculate the relative significance in relation to pollution potential^30^. In DRASTIC methods, the factor range of relative significance is evaluated on a scale of 1 (least significant) to 10 (most significant), except for the net recharge factor, which has a scale of 1 to 9.

**Table 2:** Weights and modified ratings for the pesticide DRASTICL model^30,42,43^.

Factor		Factor weight	Standard rating ranges	New rating ranges
Depth to groundwater	D	5	1–10	1–9
Net recharge	R	4	1–9	1–9
Aquifer media	A	3	1–10	1–9
Soil media	S	5	1–10	1–9
Topography slope	T	3	1–10	1–9
Impact of vadose zone	I	4	1–10	1–9
Hydraulic conductivity	C	2	1–10	1–9
Land Use	L	5	1–10	1–9

Rating values were established using an ordinal scale; however, assigning range values from 1 to 10 to differentiate the relative importance between ranges has been found to be contrary to what can be truly comprehended, as the human brain can only categorize effects on a scale of 1 to 9, which is known as the scale of intensity^23,30,52^. Hence, standard index-based methods tend to overestimate resulting scores by using 10 different categories when in reality only 9 categories are comprehensible by the human brain. In this study, the method uses a range scale from 1 to 9 to address the aforementioned limitation. The new rating value ranges are shown in Table 2.

#### Depth to water table (D)

**Figure 3:**
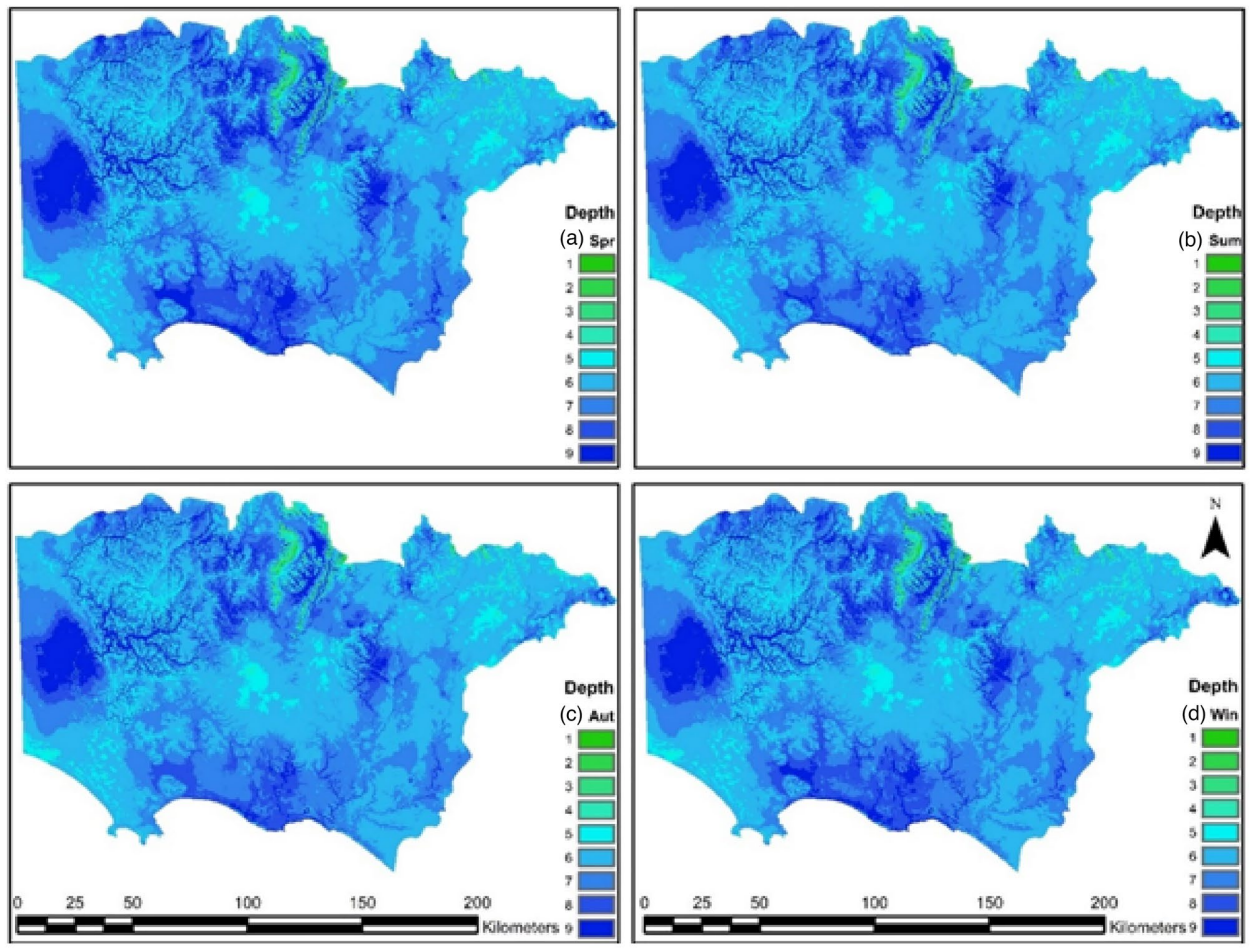
Seasonal groundwater depth (Ds) rating values for (**a**) spring, (**b**) summer, (**c**) autumn, (**d**) and winter; rating values shown on the right side of the maps.

Seasonal groundwater depths (D_S_) are shown in Fig. 3 a, b, c and d. In this study, space–time groundwater elevation maps from Peterson and Western^47^ were adopted. This approach is an extension of Costelloe et al.^53^ and Peterson et al.^54^. The maps were derived at a monthly time-step from 1 January 1980 to 1 August 2014. The maps were derived using an advanced multivariate geostatistical R package named HydroMap (https://github.com/peterson-tim-j/HydroMap). The approach allows the inclusion of topographic form, land surface elevation, coastline, and the physical constraint of the land surface elevation on the water table elevation. Furthermore, the factors within these predictors and the standard kriging factors (variogram range, sill and nugget and search radius) were derived using formal maximum likelihood estimation. Input data for kriging were derived from the HydroSight (http://peterson-tim-j.github.io/HydroSight/) time-series analysis of each groundwater hydrograph^55^. Groundwater hydrograph errors and outliers were omitted and temporally interpolated to a monthly time step using Peterson and Western^47,53^. In the aquifers of the study site where the differences in depth ranges are not large enough, a new range classification is suggested in Table 3. Seasonal depths are proposed and categorized by assigning each range a rating value from 1 to 9.

**Table 3:** Groundwater depth (Ds) rating values.

Depth to surface (m)	Rating value
0.00–0.50	9
0.50–1.50	8
1.50–3.50	7
3.50–7.00	6
7.00–15.0	5
15.0–25.0	4
25.0–40.0	3
40.0–85.0	2
> 85	1

#### Net recharge (R)

**Figure 4:**
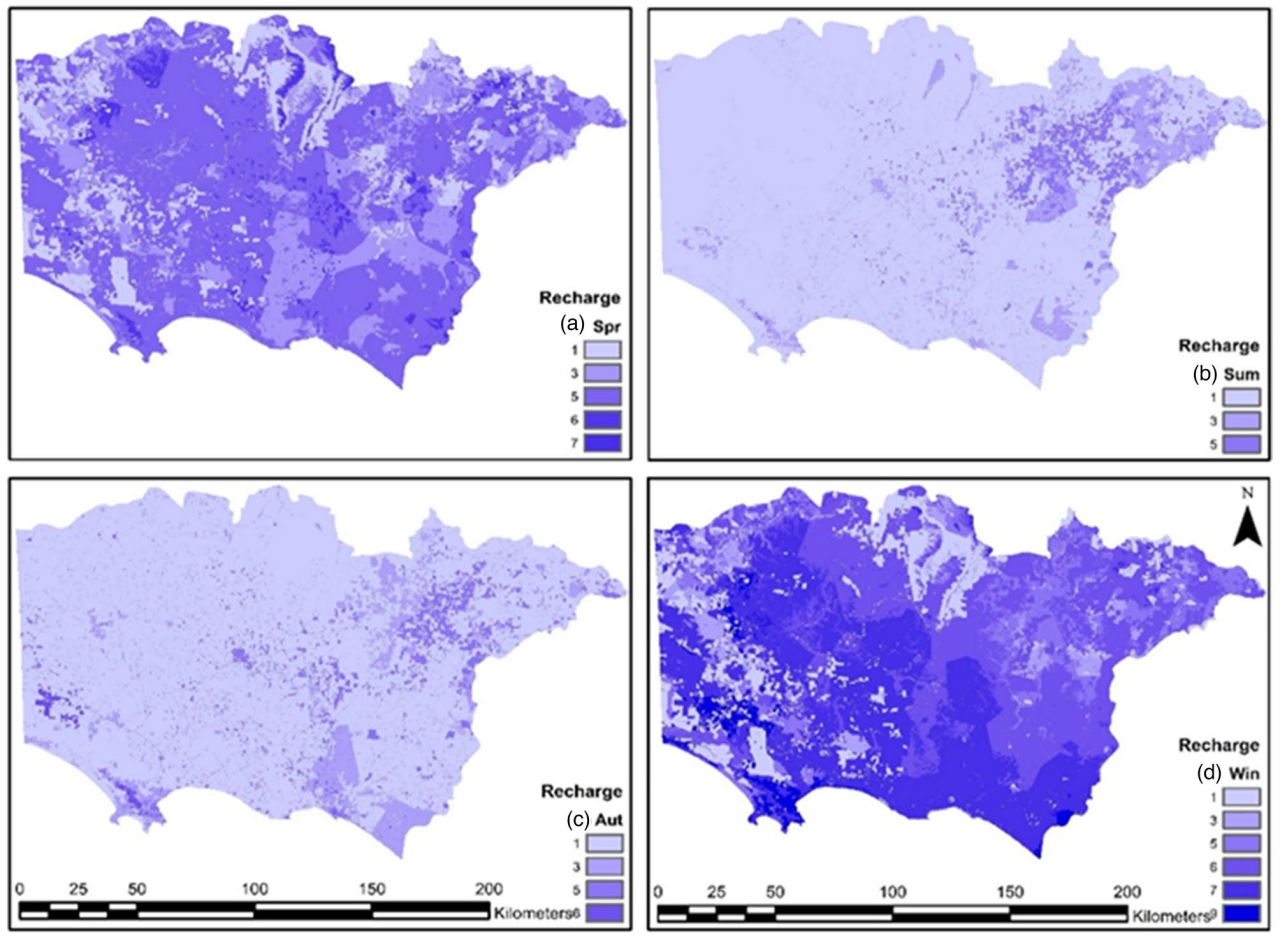
Seasonal net recharge (R) rating values for (**a**) spring, (**b**) summer, (**c**) autumn, and (**d**) winter; rating values shown on the right side of the maps.

This study uses recharge values from a transient groundwater model^45^ using monthly recharge data. The calibrated results for the steady-state and transient model normalized RMS errors were 2.47% and 2.24%, respectively. MODFLOW inputs were provided by Biosym within the Ensym model^45^. The method used to calculate recharge is described in the Australian groundwater modelling guidelines^56^. Seasonal recharge values were plotted in ArcMap 10.4.1 and categorized using values from Table 4. The groundwater recharge factor offers significant information to the model, as water recharge is the major driving mechanism for pollution transport^57^. Additionally, it provides specific information for both spatial and temporal variability. Monthly recharge values for the period from 1987 to 2017 were considered for this analysis. Seasonal recharge maps are shown in Fig. 4.

**Table 4:** Net recharge (Rs) rating values.

Recharge Range (mm)	Rating value
0–1	1
1–3	3
3–7	5
7–14	6
14–22	7
> 22	9

#### Aquifer media (A)

**Figure 5:**
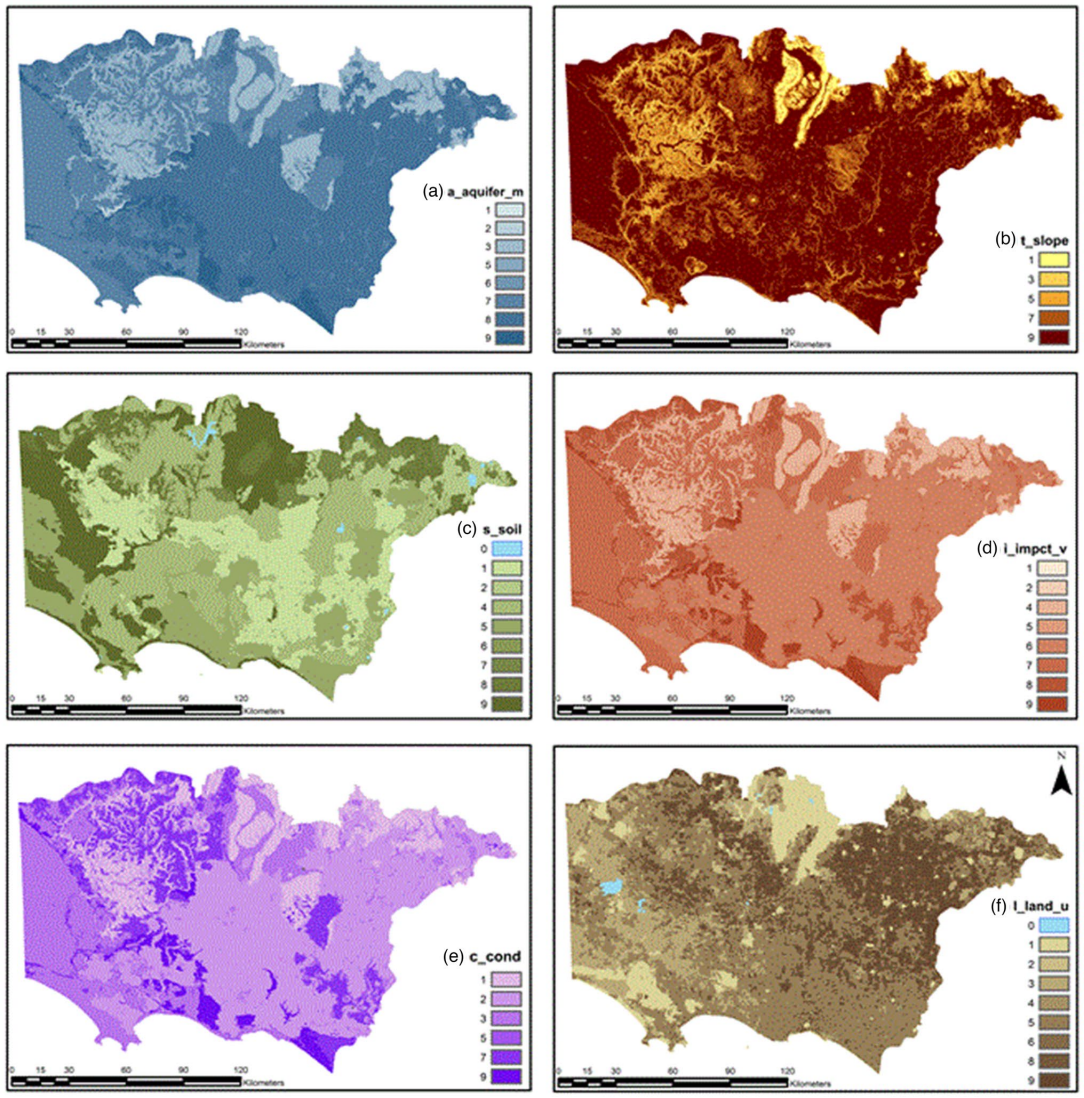
Rating values for (**a**) aquifer media, (**b**) topographic slope, (**c**) soil, (**d**) impact of vadose zone, (**e**) hydraulic conductivity, and (**f**) land use. Rating values shown on the right side of the maps.

Aquifer media (A) serves as a conduit for aquifer pollution; the larger the grain size and the more fractures there are in the aquifer, the greater the pollution potential^23^. The aquifer medium or media are responsible for controlling the route and path length for a contaminant to flow; the larger the porosity of the aquifer is, the lower the attenuation capability, and therefore, the larger the pollution potential. Aquifer configuration materials were obtained from the Victorian Aquifer Framework (VAF)^58^. The lithological units were categorized with new rating values from 1 to 9 and are presented in Table 5. Next, values were modified from Aller et al.^23,43^ and adapted to the Victoria geological units (see Fig. 5a).

**Table 5:** Aquifer media (A) rating values^17^.

Aquifer media	Rating value
Massive shale	1
Methamorphic/igneous	2
Weathered metamorphic igneous	3
Thin bedded sandstone, limestone	
Shale sequences	5
Massive sandstone	5
Massive limestone	5
Sand and gravel	7
Basalt	8
Karst limestone	9

#### Soil media (S)

Soil media is the upper portion of the vadose zone; it has significant biological activity, and it is considered the upper weathered zone of the Earth, averaging six feet (1.8 m) or less^23^. Soil media control recharge and contaminant attenuation processes such as filtration, biodegradation, sorption, and volatilization^23^. The upper portion of the soil configuration is used for this factor. Data were extracted from the Atlas of Australian Soil (ASRI), and the variable that represents the topmost layer is (TEXT_TOP) within the Australian Soil Atlas Dataset. The categorization of the Australian soil texture is found in the glossary of soil terms and in “Estimation of Soil Properties using the Atlas of Australian Soils”^59^. A rating for soil texture groups is presented in Table 6, and the map for the soil media is shown in Fig. 5c.

**Table 6:** Soil (S) rating values.

Texture group	Texture grade	Rating value
Sands	Sand	9
	Clayey sand	9
	Loamy sand	9
Sandy loams	Sandy loam	8
	Fine sandy loam	8
	Light sandy loam	8
Loams	Loam	7
	Loam, fine sandy	7
	Silt loam	6
	Sandy clay loam	6
Clay loams	Clay loam	5
	Silty clay loam	4
	Fine sandy clay loam	4
Light clays	Sandy clay	3
	Silty clay	3
	Light clay	2
	Light medium clay	2
Clays	Medium clay	1
	Heavy clay	1

#### Topography slope (T)

Topography refers to the slope and slope variability in the land surface; it controls the probability of a contaminant running off or remaining on the surface in a specific area long enough for it to enter the aquifer^23^. The topography is described in terms of slope percentage^41^, and the topography slope map was constructed from the digital elevation model (DEM) using ArcMap10.4.1, with a cell size of 50 m × 50 m. The slope was then categorized according to the slope rating values, as shown in Table 7. Figure 5b illustrates the slope % for the study area.

**Table 7:** Topography slope (T) rating values.

Slope %	0–2	2–6	6–12	12–18	> 18
Rating value	9	7	5	3	1

#### Impact of vadose zone (I)

The vadose zone is defined as the zone above the water table that is unsaturated; it lies below the soil horizon and above the water table^30^. In the vadose zone, attenuation processes such as biodegradation, neutralization, filtration, chemical reaction, volatilization, and dispersion are likely to decrease as depth increases^23^. The rating for the vadose zone map was created using the groundwater flow system (GWFS) and the material conformation for each of the aquifer types; conformation material is described in the Victorian aquifer framework^49^. Table 8 shows the vadose zone ranges and rating values proposed for the Pesticide DRASTICL model. Figure 5d shows the impact of the vadose zone map categorized with the rating values of the modified Pesticide DRASTICL model.

**Table 8:** Impact of vadose zone (I) ratings.

Name	Range	Rating value
Sand	6–9	7
Bedded limestone, sandstone, shale	4–8	6
Silt, sand,	6–9	8
Sand, silt	4–8	8
Gravel, salt, silt, silt, clay	4–8	7
Karst	9	9
Silt and clay	1–2	1
Marine deposits karst	8–9	9
Sandstone gravel, sand	4–8	5
Basalt	2–9	8
Sand and gravel silt, clay	4–8	6
Metamorphic/Igneous	2–8	4

#### Hydraulic conductivity of the aquifer (C)

Hydraulic conductivity (C) is the aquifer’s material capability to transmit water; it controls the flow rate of groundwater at a given hydraulic gradient, intergranular porosity, tectonic lineaments, and bedding planes^23^. Similarly, contamination is controlled by the flow rate of groundwater^41^. Furthermore, C controls the contamination movement in the aquifer^23^. Within index-based methods, C is denoted as the factor with the highest error associated with its estimation^60^. The factor rating for C was obtained from two sources. The first is an inferred value from various reports included in the GH GWFS by Dahlhaus et al.^48^ The second is the data containing conductivity values from a groundwater transient model by SKM^45^. In this study, k values (m/d) were used from SKM^45^. A new rating scale is assigned to each range of hydraulic conductivity, as shown in Table 9. A rating map for C is shown in Fig. 5e.

**Table 9:** Hydraulic conductivity (C) (m/d) rating values.

(k) Range	Rating
81.50 +	9
40.75–81.50	7
28.53–40.75	5
12.23–28.53	3
4.08–12.23	2
0.00–4.08	1

#### Land use (L)

Changes in land use induce the application of agricultural chemicals, industrial waste spills and landfill leachate, which can potentially leach into groundwater. L is a decisive and inducing factor of aquifer contamination thorough anthropogenic activities^60^. Research shows that specific methods that integrate information about land use perform better than intrinsic approaches^61^. Specifically, land use provides additional information on the use of the actual landscape, the type of cropping and crop rotation, which can be used to identify the amount and type of agricultural products used in the catchment^61^. This approach has been successfully applied in India^43^, Iraq, Saudi Arabia^62,63^, Greece^64^, Portugal^25,65^, United Kingdom, United States^23,66^ and Australia^67^.

Data from 2016 were downloaded from Agriculture Victoria (2018)^51^, and we selected the secondary land use cell of the Victorian land use information system (VLUIS) to assign new rating values from 1 to 9, as shown in Table 10. Figure 5f shows the land use rating map. Additionally, it shows the land use rating values for different land uses. Next, a raster file was created including the new ratings.

**Table 10:** Land use rating values.

Secondary_V7	Rating
1.1 Nature conservation	1
2.1 Grazing native vegetation	3
2.2 Production forestry	4
3.2 Grazing modified pastures	3
3.3 Cropping	9
3.4 Perennial horticulture	4
5.2 Intensive animal husbandry	8
5.3 Manufacturing and industrial	7
5.4 Residential and farm infrastructure	6
5.5 Services	3
5.7 Transport and communication	2
5.8 Mining	8
5.9 Waste treatment and disposal	7

#### Multivariate statistical assembly of seasonal groundwater vulnerability

In standard DRASTIC methods, GWV has been estimated as a fixed or static value. However, such methods have failed to depict the impacts of temporal variations. Vulnerability can be affected by several factors, such as groundwater extraction, temporal variations in precipitation and evapotranspiration rates, temporal variations in groundwater depths, and temporal variations in groundwater recharge.

To make the vulnerability index sensitive to those changes, the proposed multivariate statistical analysis integrates time variations in depth and recharge while estimating seasonal GWV. This was achieved by estimating a GWV value for each season (spring, summer, autumn and winter). For estimating time-variant values, Eq. (1) was transformed into four different GWV estimations as presented below.

2 $$ {\text{I}}_{{{\text{DSpr}}}} = \, \left( {{\text{Dr}}_{{{\text{Spr}}}} *{\text{Dw}}_{{{\text{Spr}}}} + {\text{ Rr}}_{{{\text{Spr}}}} *{\text{Rw}}_{{{\text{Spr}}}} } \right) + {\text{ Ar}}*{\text{Aw }} + {\text{ Sr}}*{\text{Sw }} + {\text{ Tr}}*{\text{Tw }} + {\text{ Ir}}*{\text{Iw }} + {\text{ Cr}}*{\text{Cw }} + {\text{ Lr}}*{\text{Lw}} $$

3 $$ {\text{I}}_{{{\text{DSum}}}} = \, \left( {{\text{Dr}}_{{{\text{Sum}}}} *{\text{Dw}}_{{{\text{Sum}}}} + {\text{Rr}}_{{{\text{Sum}}}} *{\text{Rw}}_{{{\text{Sum}}}} } \right) + {\text{ Ar}}*{\text{Aw }} + {\text{ Sr}}*{\text{Sw }} + {\text{ Tr}}*{\text{Tw }} + {\text{ Ir}}*{\text{Iw }} + {\text{ Cr}}*{\text{Cw }} + {\text{ Lr}}*{\text{Lw}} $$

4 $$ {\text{I}}_{{{\text{Daut}}}} = \, \left( {{\text{Dr}}_{{{\text{Aut}}}} *{\text{Dw}}_{{{\text{Aut}}}} + {\text{ Rr}}_{{{\text{Aut}}}} *{\text{Rw}}_{{{\text{Aut}}}} } \right) \, + {\text{ Ar}}*{\text{Aw }} + {\text{ Sr}}*{\text{Sw }} + {\text{ Tr}}*{\text{Tw }} + {\text{ Ir}}*{\text{Iw }} + {\text{ Cr}}*{\text{Cw }} + {\text{ Lr}}*{\text{Lw}} $$

5 $$ {\text{I}}_{{{\text{DWin}}}} = \, \left( {{\text{Dr}}_{{{\text{Win}}}} *{\text{Dw}}_{{{\text{Win}}}} + {\text{ Rr}}_{{{\text{Win}}}} *{\text{Rw}}_{{{\text{Win}}}} } \right) + {\text{ Ar}}*{\text{Aw }} + {\text{ Sr}}*{\text{Sw }} + {\text{ Tr}}*{\text{Tw }} + {\text{ Ir}}*{\text{Iw }} + {\text{ Cr}}*{\text{Cw }} + {\text{ Lr}}*{\text{Lw}} $$

whereI_D (Spr, Sum, Aut, Win)_ = seasonal vulnerability indexDr _(Spr, Sum, Aut, Win)_ = depth ratings per seasonDw _(Spr, Sum, Aut, Win)_ = depth weights per seasonRr _(Spr, Sum, Aut, Win)_ = recharge ratings per seasonRw _(Spr, Sum, Aut, Win)_ = recharge weights per season

After calculating the seasonal vulnerability index Eqs. (2–5), we addressed the estimation of a new set of factors weights with the purpose for eliminating the correlation between factors^25^. This was achieved by separating DRASTICL factors into two hydrogeological groups: the dynamic group (D and R) and the static group (A. S, T, I, C and L). For both groups, an independent CA was performed, and a new set of upscaled factor weights was obtained for each season. CA deals with the subjectivity bias from using expert opinion for allocating factor weights.

This approach to data treatment (static and dynamic factors) is different from the standard index-based methods, which integrate all factor weightings in one multivariate analysis. CA has been selected because it has been shown to be the most adequate method for transforming the interrelated DRASTIC factors into uncorrelated DRASTICL vectors^25^.

First, the dynamic factors D, R plus a dummy factor (Q) with a constant value of 5 are resampled in ArcMap 10.4.1 using a fishnet at regular intervals of 5 km. The purpose of the sampling is to extract factor rating values at each point. Next, another point resampling is performed for each of the static factors A, S, T, I, C, and L.

Second, a CA is performed to calculate vector loadings for each group. In the case of the dynamic group, a dummy dataset Q with a mean of 5 in rating value is used to assist in the calculation of the vector loadings as CA can only be performed with more than two factors. CA is also performed for the static hydrogeological group using the A, S, T, I, C and L factors. From both groups, factor loading values are derived from the CA, new vector loadings are obtained and represent the uncorrelated value compared to other factors. Such values are called dynamic vectors (D, R and Q loadings) and static vectors (A, S, T, I, C, and L loadings).

Finally, each vector loading is rescaled to the DRASTICL weighting values (1–5) using the harmonization formula^25^, as shown in Eq. (6). These new sets of weights are called seasonal vector DRASTICL factor weights.

6 $$ \begin{gathered} v_{j}^{*} = \frac{{W_{j.max} {\text{x }}v_{j.max} - \left( {W_{j.max} - W_{j.min} } \right) {\text{x }}\left( {v_{j.max} - v_{j} } \right)}}{{v_{j.max} }} \hfill \\ 1 \le j \le p \hfill \\ \end{gathered} $$

wherew_j.max_ = max factor weight in Pesticide DRASTICw_j.min_ = min factor weight in Pesticide DRASTICvj = factor loadings

The CA was computed using R, an open-access software^25^. New loading values for vector1 (V1) are calculated as mentioned earlier. The calculated loading values from the CA cannot be compared to the Pesticide DRASTICL weighting values because the loadings are presented on a different scale. From Eq. (6), a new set of re-coordinated weighting values from new vector-DRASTICL factor loadings were calculated. Each factor group was treated as a separate multivariate statistical dataset. Re-coordinated weighting values are known as vector-DRASTICL factor weights^25^.

#### Estimating the time-variant Pesticide DRASTICL vulnerability

To improve the outputs of the Pesticide DRASTICL method, factor ratings were reduced to a maximum value of 9, and an independent multivariate CA was applied to both factor groups. After rating categorization, a resample is made using a fishnet of 5 × 5 km to obtain point values from each factor. The fishnet is the base for digital sampling of each of the factors included in the CA. The harmonization of scales method was applied to the resulting CA vector loading values, re-coordinating them from 1 to 5. As a result, seasonal Pesticide DRASTICL weighting values were used to estimate seasonal GWV.

The seasonal estimation of the GWV was quantified by using the corresponding seasonal factor weights. Eight raster layers from DRASTICL factors were used to calculate the seasonal I_D_. The results were mapped in the study area for each of the seasons.

Different values for vulnerability were expected for each season. The vulnerability values could be similar in places where the levels of depth and water recharge had small or no changes, indicating that the aquifer hydrogeological properties are less sensitive to climatic changes. Conversely, vulnerability values should be higher where depth and recharge showed large seasonal changes in depth and recharge.

#### Consent to participate

By this means, we the authors give explicit consent to participate in the submission of this scientific paper.

## Results

### Correspondence analysis and time-variant groundwater vulnerability

**Figure 6:**
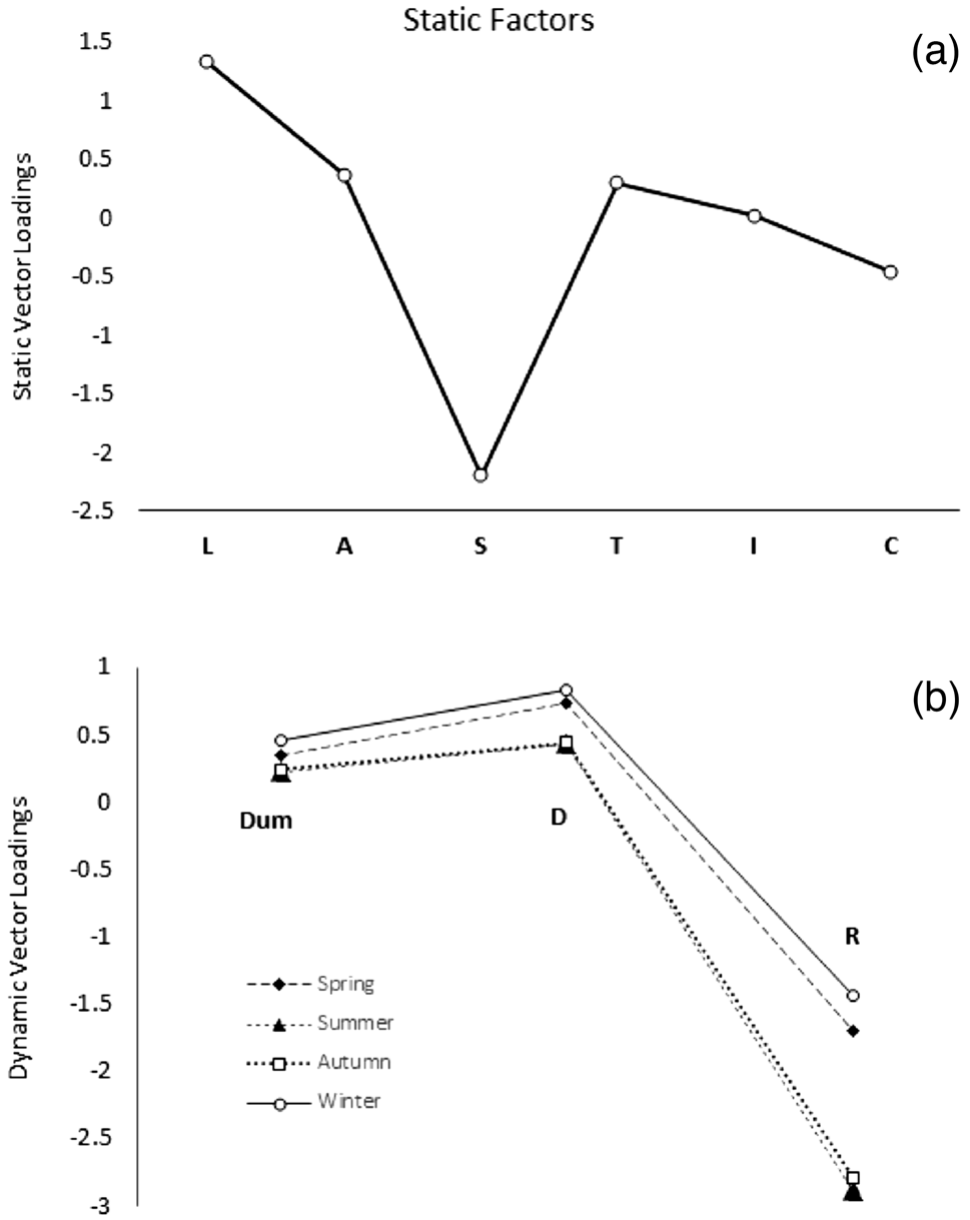
(**a**) Distribution of CA V1 loadings for static factors and (**b**) distribution of CA V1 loadings for dynamic factors.

Vector loadings for the static groups L, A, S, T, I, and C are plotted in Fig. 6a, where the largest absolute value in loadings corresponds to S. Figure 6b shows the results for common vector loadings from the dynamic vector 1 D, vector 2 R and vector 3 Dummy (Dum) mapped for spring, summer, autumn and winter.

The application of CA to data for the seasonal group resulted in the confirmation that the use of a dummy dataset (Dum) does not impact the value of the cumulative percentage of the system variance. It can be seen that Factor R has a larger loading compared to D. As shown in Fig. 6a, there are differences in the CA loading values for both groups; the larger the absolute value, the higher the non-correlation between factors. Such variables are called explicative factors; for the non-explicative factors, the values were the lowest^42^. In the case of the dynamic factor group, Factor R has a higher weight than Factor D. This could be explained because recharge is the driving hydrogeological process through which aquifer pollution occurs; the higher the recharge, the higher the chances for a pollutant to be transported to the water table^57^.

**Figure 7:**
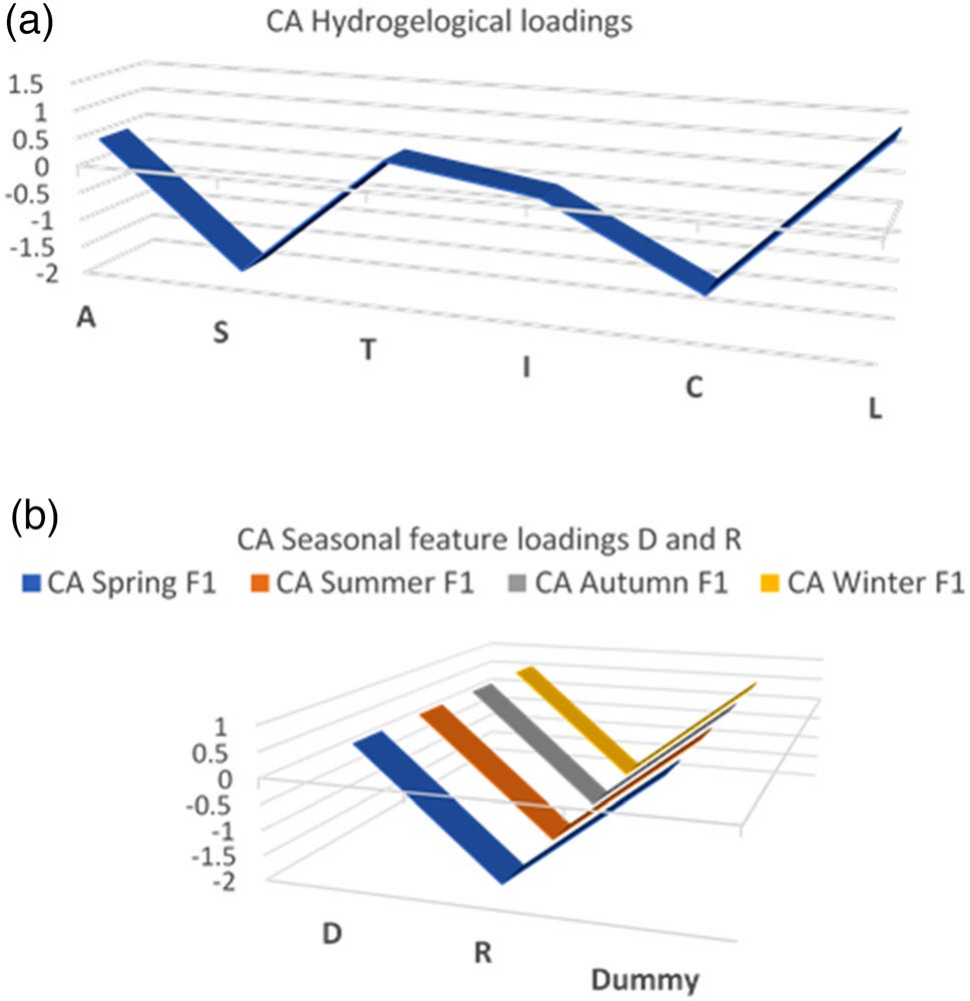
(**a**) Results of dynamic CA eigenvalues. (**b**) Plot of D, R and Dum loadings for spring, summer, autumn and winter.

Figure 6a shows the distribution of eigenvalues and the cumulative percentage of the system variance for common vectors V1, V2, and V3. Two explicative factors for the dynamic group vector 1 D and vector 2 R can explain 100% of the system variance. Figure 7b shows the loading values for V1 and V2 plotted for each season, and a clear difference in the loadings for R can be seen. Table 11 shows the cumulative percentage of the system variance and eigenvalues for each of the seasons. It can be seen that Dum has synthetic eigenvalues of zero and that both D and R can explain 100% of the system variance. Table 12 shows the results for dynamic CA factor loading values, which show that in absolute values, Factor R has higher loadings than Factor D.

**Table 11:** Seasonal CA eigenvalues and cumulative % of system variance.

Season	Eigenvalue			Cumulative % of system variance		
	D	R	Dum	V1	V2	V3
Spring	0.0354	0.0063	0.0	88.89	100	100
Summer	0.0456	0.0075	0.0	89.51	100	100
Autumn	0.0566	0.0068	0.0	89.39	100	100
Winter	0.0387	0.0054	0.0	88.49	100	100

**Table 12:** Seasonal factor loadings.

Season	Factor loads		
	D	R	Dum
Spring	0.732	−1.707	0.345
Summer	0.432	−2.891	0.220
Autumn	0.439	−2.802	0.238
Winter	0.832	−1.444	0.463

**Figure 8:**
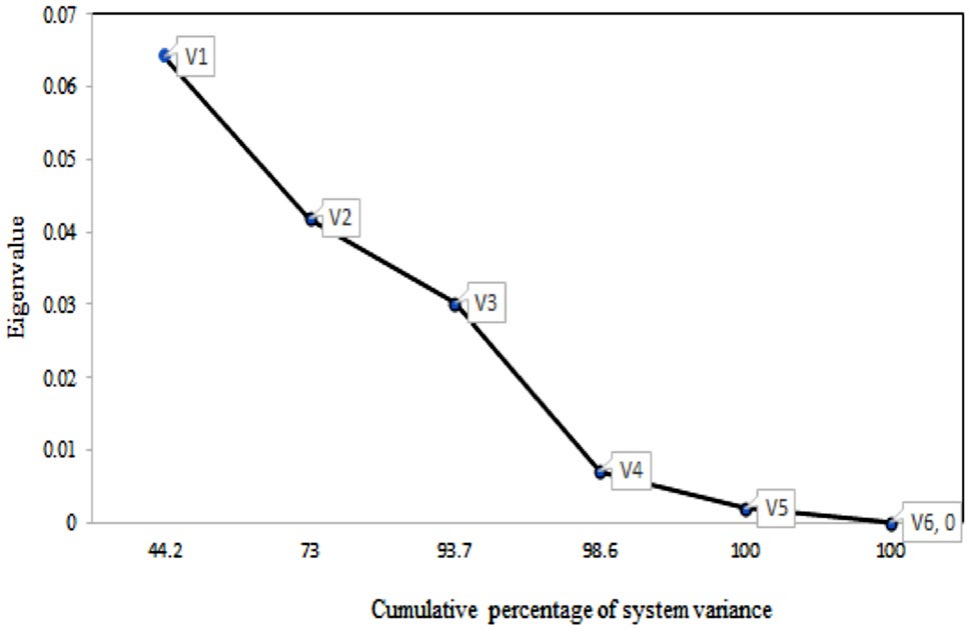
Results of CA for static factors. SCREE plot of common vectors V1, V2, V3, V4, V5 and V6.

**Figure 9:**
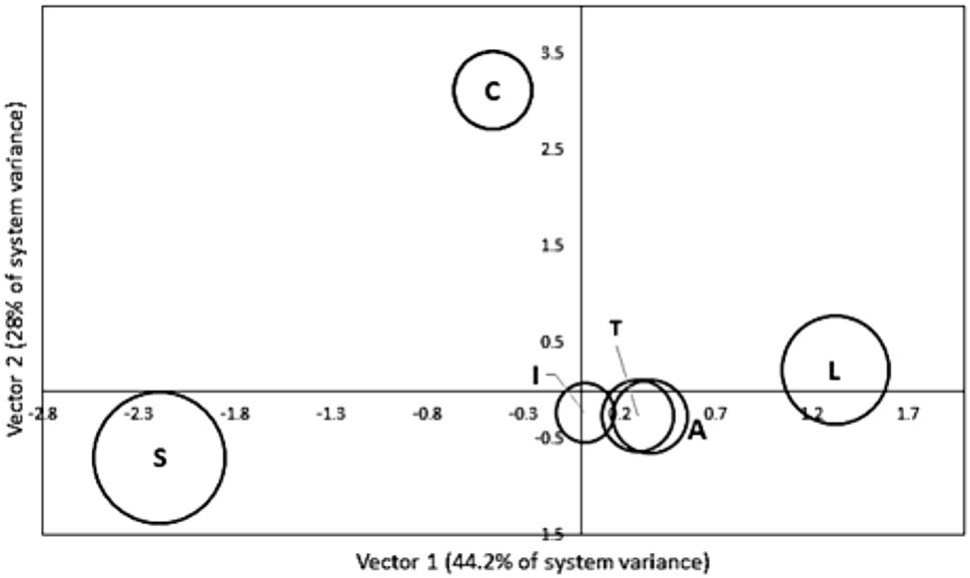
Results of CA for static factors. Cartesian projection of vector loadings V1, V2, V3, V4, V5 and V6.

The second CA is performed for the static group (Fig. 7a). Table 13 shows the results of the CA static eigenvalues and cumulative percentage of variance. Figure 8 shows the results of CA for the static factors, and common vectors V1, V2, V3 and V4 account for the cumulative percentage variance at approximately 98.6%. Figure 9 shows the distribution of static CA V1 and V2 loadings, which shows that vectors S, L and C are the explicative factors of the static system. Vectors S, L and C are the ones with the highest loadings and the highest distances from the centroid. Although the results from static CA suggest that principal static vectors V1, V2 and V3 can explain up to 93.7% of the system variance (see Fig. 8) and that using only these three factors should be enough to derive the vulnerability index, all static factors were considered for this study under the premise that each one is a significant explicative factor that contributes different types of information to the time-variant Pesticide DRASTICL vulnerability index.

**Table 13:** Static CA eigenvalues and cumulative % of system variance.

Factor	Static vectors					
	L	A	S	T	I	C
Vector	V1	V2	V3	V4	V5	V6
Eigenvalue	0.0642	0.0418	0.030	0.007	0.0019	0.00
% Cum. var	44.2	73	93.7	98.6	100	100

New seasonal vector DRASTICL weights are presented in Tables 14 and 15 as a result of the harmonization of scales using the loading values for each of the factors. Such weights will be used in the calculation for the seasonal Pesticide DRASTICL vulnerability. The new vulnerability is referred to as the seasonal vector DRASTICL index and is represented as I_DSpr_, I_DSum_, I_Daut_, and I_DWin_.

**Table 14:** Static vector DRASTIC factor weights.

Factors	${\mathrm{v}}_{\mathrm{j}}^{*}$
L	3.42
A	1.66
S	5.00
T	1.54
I	1.04
C	1.83

**Table 15:** Dynamic vector DRASTIC factor weights.

	$${\mathrm{v}}_{\mathrm{j}}^{*}$$	
	D	R
Spring	2.72	5.00
Summer	1.60	5.00
Autumn	1.63	5.00
Winter	3.30	5.00

**Figure 10:**
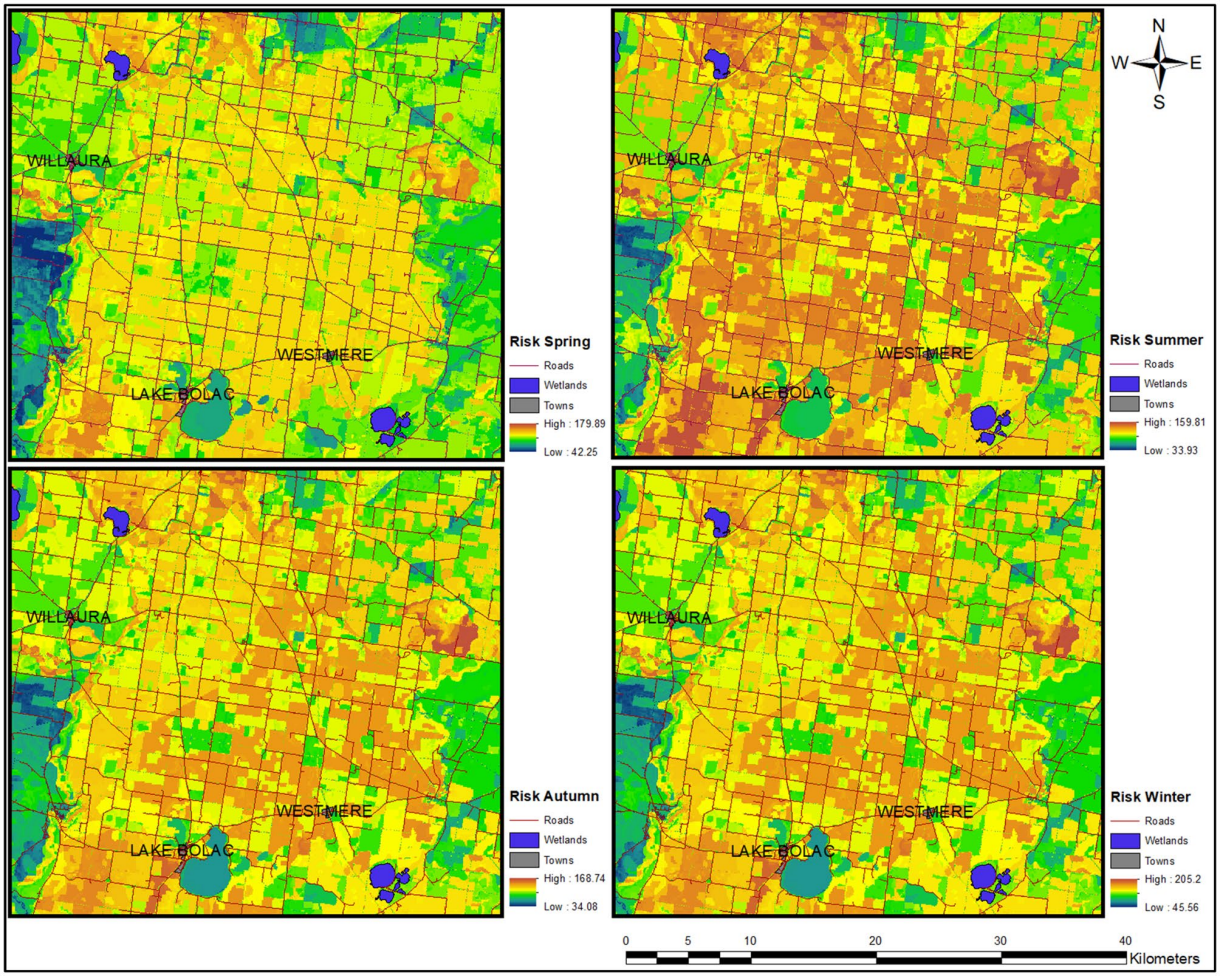
Seasonal vulnerability estimations for the central region of the GH Catchment Management Authority, Victoria, Australia. Left, GWV for spring and autumn. Right, GWV for summer and winter.

From Fig. 10, it can be observed that there is a dimensionless scale for each season that should be approached as an independent representation of vulnerability. The results also showed that the risk was highly impacted by factors R, D, and L. The model was capable of estimating seasonal changes in vulnerability, with summer presenting the highest risk of contamination to groundwater in this specific area.

### Comparison of vulnerability predictions with observed pollutants

**Figure 11:**
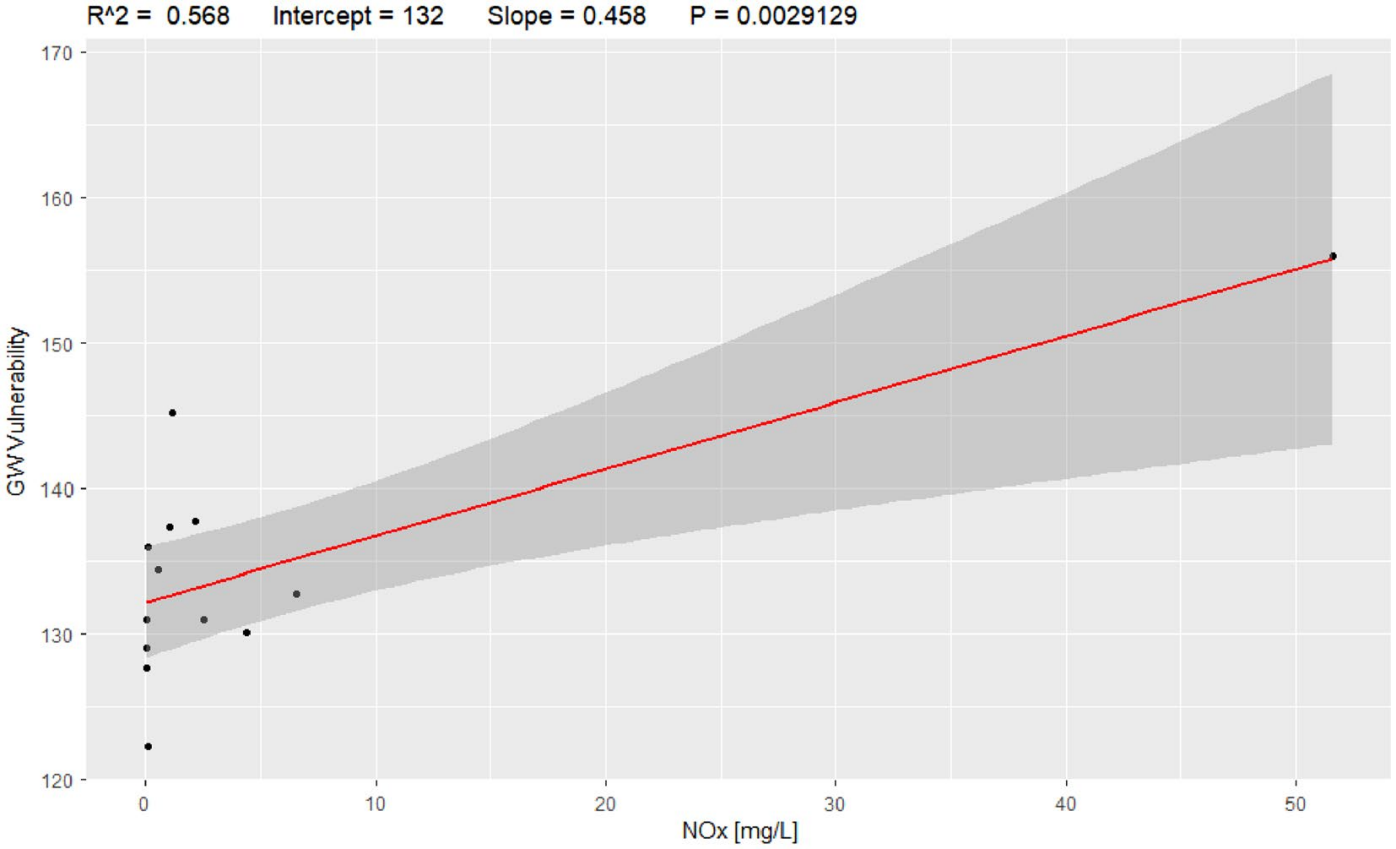
Model correlation between vulnerability values and NOX [mg/L], R^2^ = 0.568.

**Figure 12:**
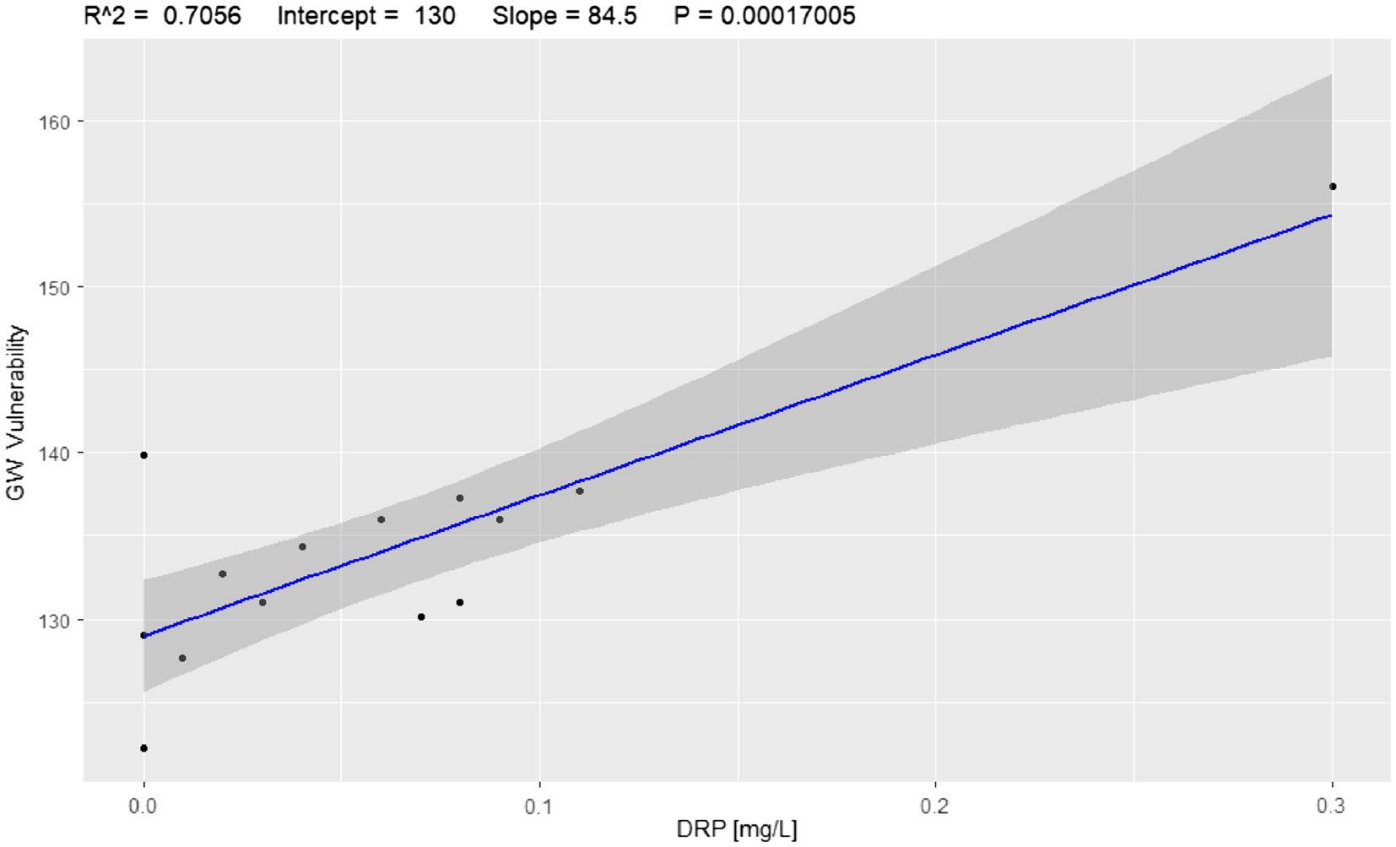
Model correlation between vulnerability values and DRP [mg/L], R^2^ = 0.70.

A comparative evaluation between the model and observed pollution was performed for nitrates (NO_3_^−^) + nitrites (NO_2_^−^) as NOX and dissolved reactive phosphorus (DSR) as phosphorus. Using the vulnerability map for winter, a field survey was designed and deployed at the study site for 2017 and 2018. Using the results from the model, 18 bores were sampled and analysed across the study site, as shown in Fig. 2. Figures 11 and 12 show the correlation coefficients (R^2^ = 0.568 and 0.7056) for nitrogen and phosphorous, respectively. It can be observed that phosphorus outperforms nitrogen in evaluating the performance of the model. However, it should be considered that phosphorus showed fewer occurrences in groundwater and that the concentrations are lower compared to nitrogen. Researchers commonly use nitrogen to evaluate standard DRASTIC model performance.

## Discussion

This study presents a novel approach to estimate a time-variant GWV as an alternative to a previously published static vulnerability^31^. Each seasonal vulnerability estimation is the representation of different conditions in the aquifer that reflects physical changes in the environment and should be interpreted accordingly. The resulting seasonal GWV is an independent estimation for each of the seasons. The qualitative categorization of vulnerability (very high, high, medium, low and very low), as mentioned in most previous research, may lead to the loss of valuable information for the user. In this study, a scale provides better insights into seasonal changes. The higher the vulnerability value on this scale, the higher the risk of contamination to groundwater.

Vulnerability values ranged from 42.25–179.89, 33.93–159.81, 34.08–168.74, and 45.56–205.20 for spring, summer, autumn, and winter, respectively. A lower vulnerability value was observed in summer, which is the season where less water is available for the aquifer due to less precipitation and high temperatures, producing the highest evapotranspiration rates and having a direct impact on the groundwater net recharge. Conversely, the highest vulnerability value occurred in winter, where the environmental conditions are the opposite of those in summer. This means that the vulnerability scale behaviour is consistent with the behaviour of the transient model in terms of groundwater net recharge. Two studies have reported seasonal estimations of the GWV for the pre-monsoon, monsoon and post-monsoon seasons^61,68,69^. However, those studies failed to comprehensively assess the relative weights for contributing factors for each season and considered all factors to be static over time. The results from this study suggest that the weights vary between seasons. The novelty of the proposed time-variant Pesticide DRASTICL model focuses on the separation of the factors (static and dynamic) and in the comprehensible calculation of the factors’ weights.

The seasonal CA suggests that the highest weight corresponds to the dynamic Factor R, followed by the static Factor L, while D showed a significant variation in weighting values between seasons, being highest in winter and spring. That is, in the season with the highest precipitation, D is weighted higher than in summer with the lowest weighting value.

In groundwater pollution surveying, it is common for researchers to use surrogate pollutants to predict the likelihood of pesticide pollution, as pesticide analysis is expensive and requires sophisticated laboratory facilities. Instead of analysing a large number of groundwater samples for pesticides, we selected a smaller number of samples that could show high concentrations of NOX and/or DRP for potential pesticide contamination. We also changed the original weights recommended by Allert et al.^30^ using a correspondence analysis that derived new weights based on how sound a factor is compared to the others.

Phosphors showed a better correlation with the predictions of the model. A fundamental step in standard index-based methods is the calibration process^70^; however, in this study, there are no seasonal data available for the seasonal calibration step, and this has been identified as a major gap requiring consistent effort and investment. We suggest that the calibration of the proposed time-variant Pesticide DRASTICL model should be carried out with seasonal nitrogen and/or phosphorus concentration data and that the continuous use and calibration of the model will lead to better performance.

## Conclusions

In current index-based methods, the vulnerability index is mapped as a fixed scale assuming that such values do not change in the study area throughout the year. These methods ignore time variations in vulnerability. This study has addressed this knowledge gap by categorizing the influencing factors into two hydrogeological groups (static and dynamic) to estimate a time-variant groundwater vulnerability. In this approach, a Pesticide DRASTICL model was coupled with a double correspondence analysis to develop a seasonal vulnerability index.

The results of the model indicated that the vulnerability index varies between seasons and highly depends on the two dynamic factors, groundwater recharge and groundwater depth. We found that groundwater recharge and soil media are the explicative factors in the seasonal quantification of GWV. The model results showed a good correlation with the observed nitrogen and phosphorus levels. However, the model needs to be a recursive system of use, testing, and calibration, as there is not enough seasonal data. The quantification of temporal and spatial variations in the GWV will provide additional information to farmers and water administrators to better manage groundwater pollution prevention programs. It will also assist with the use of fertilizers and pesticides in agricultural areas.

## Data Availability

The data that support the findings of this study are available from the corresponding author upon reasonable request.

## Code availability

The code that supports the findings of this study is available from the corresponding author upon reasonable request.
